# The Effect of Minerals and Hormones on the Nutrients in Chinese Fir Leaves and Seed Set

**DOI:** 10.3390/plants14060887

**Published:** 2025-03-12

**Authors:** Yu Duan, Linying Zhao, Daiquan Ye, Jian Zhou

**Affiliations:** 1 Co-Innovation Center for Sustainable Forestry in Southern China, College of Forestry, Nanjing Forestry University, Nanjing 210037, China; duanyu@njfu.edu.cn; 2 Co-Innovation Center for Sustainable Forestry in Southern China, College of Life Sciencese of Biology and the Environment, Nanjing Forestry University, Nanjing 210037, China; zhaolinying76@gmail.com; 3 Fujian Yangkou State Owned Forest Farm, Nanping 353211, China; rain@warmbook.cn

**Keywords:** Chinese fir, seed-setting characteristics, foliar fertilization, nitrogen, phosphorus, potassium

## Abstract

To investigate the impacts of various foliar fertilization levels on the carbon, nitrogen, phosphorus, and potassium content in Chinese fir cuttings, along with their ecological stoichiometry ratios, we selected pruned dwarf Chinese fir clones exhibiting different seed-setting abilities as our subjects. These clones were categorized into high-yield (group A), middle-yield (group B), and low-yield (group C) categories for nutrient assessment. Employing nine treatments of diverse fertilizers and hormones, in addition to a water control (CK), we analyzed and compared the changes in the carbon (C), nitrogen (N), phosphorus (P), and potassium (K) contents in the needles of Chinese fir clones from groups A, B, and C between 2021 and 2022. The results indicated no significant variations in the N content and C:N ratios in July among the three seed-setting characteristic Chinese fir types. However, the P content in the high-yield clones was notably higher than that in the other two types, whereas the K content was significantly lower. Following two years of continuous foliar spraying, treatments T5, T6, T8, and T9 demonstrated efficacy in enhancing the nutrient levels of branches in high-yield clones (with N content increasing by 25.07%, P content by 79.06%, and K content by 12.71%), consequently improving cone quality (as the number of cones increased by up to 256). For middle-yield clones, treatments T3, T6, and T9 exhibited promising outcomes, with respective increases in the N content, P content, and K content by 13.15%, 56.61%, and 41.31%, alongside a rise in cone number by 212. In the case of low-yield clones, the treatments T3, T4, and T5 proved effective, with increases in the N, P, and K contents by 18.54%, 36.57%, and 26.56%, respectively, as well as an increase in cone number by 82. Most treatments exhibited higher C:N ratios than the control in Chinese fir needles, whereas the N:P ratios remained below 14, indicating N limitations in the growth of Chinese fir clones. The application of N fertilizer enhanced the C:N ratios in Chinese fir needles, thereby improving nutrient absorption and utilization efficiency. Therefore, in the fertilization process of Chinese fir, tailored formulas should be employed based on the seed-setting characteristics and management objectives to achieve optimal yield enhancement.

## 1. Introduction

*Cunninghamia lanceolata* (Lamb.) hook is a fast-growing tree species that is extensively utilized in southern China [1]. *Cunninghamia lanceolata* demonstrates robust upward growth, with its main seed layer typically situated in the uppermost or upper-middle section of the tree. As the tree height continues to increase, the seed collection in Chinese fir orchards faces challenges. Consequently, the pruning and “dwarfing” of maternal trees within seed orchards to reduce seed layer height have emerged as critical technologies in the forestry seed orchard industry and significant components of forest seed base management. A technical challenge of modern seed orchard is to ensure that the pruned and dwarfed *Cunninghamia lanceolata* trees maintain an adequate number of reproductive branches to meet the yield requirements while also possessing sufficient vegetative branches to support cone or seed growth and development, thereby guaranteeing sufficient supply of the flower buds of *Cunninghamia lanceolata* for the following year’s harvest [2].

Fertilization and hormone application are crucial methods for promoting forest growth to enrich the soil nutrient levels and provide various nutrients essential for tree development, thereby regulating the nutrient balance of forests and forested areas [3]. N, a fundamental nutrient for plants, is the cornerstone of robust tree growth. The differentiation, formation, and structure of plant organs are directly related to the availability of N [4], and are intimately linked with photosynthesis [5]. Appropriate N applications can modulate budding, promote leaf development and growth, and facilitate flower bud differentiation [6]. Different N fertilizer treatments on Chinese fir seedlings have demonstrated that optimal N levels are advantageous for enhancing light energy absorption, conversion, and utilization efficiency in Chinese fir needles, thereby improving the leaf photosynthetic capacity and facilitating the synthesis of carbon assimilation products [7]. This, in turn, augments seedling growth and enhances seedling quality [8]. Appropriate P levels can contribute to robust shoot growth, efficient flower bud differentiation, and early fruit ripening, whereas P deficiency may lead to reduced flowering, fruit drop, and other adverse effects [9]. Adequate P fertilizer content can decrease the N:P ratio in Chinese fir needles, thereby enhancing the female flower bud formation and clone seed orchard yields [10]. K, an essential monovalent cation for plant growth, is widely acknowledged as a “quality element” [11]. It enhances photosynthetic intensity by improving photosynthetic pigments [12], elevates seed-setting quality by affecting photosynthesis [13], promotes protein and sugar synthesis and transport in plants, and enhances seed quality [14]. Gibberellin can promote forest tree growth and accelerate plant growth and development by promoting the division of young meristematic cells [15]. They regulate plant flowering, increase yield, break seed dormancy, promote germination, alter male–female flower ratios, and reduce flower and fruit shedding [16]. Auxins facilitate plant growth [17]; inhibit the formation of separation zones; prevent flower, fruit, and leaf drops; and promote fruiting [18]. The early varieties of foliar fertilizers were relatively limited in diversity, with less pronounced effects on yield. Subsequently, as multiple foliar fertilizers have been researched and applied, the variety of fertilizers continually expanded and updated, leading to significant advancements in foliar fertilizers and their application effects [19]. Fertilizers can only fully exert their estrogen-enhancing effects under the appropriate timing, quantities, and optimal ratios. In a fertilization experiment conducted in Chinese fir seed orchards using N, P, and K fertilizers, under balanced fertilization treatments, the thousand-seed weight, germination rate, and seed quality of Chinese fir seeds were notably higher than those under standard fertilization practices [20].

Ecological chemometrics, a scientific discipline, investigates the theory of multi-element balance and system energy cycles [21] and is a significant method for assessing material cycles and nutritional status in terrestrial ecosystems [22]. In plants, leaf carbon constitutes the most vital element in dry matter, representing approximately 38% of a plant’s dry mass [23]. The carbon to nitrogen (C:N) and P ratios in plants can indicate growth rate and nutrient utilization efficiency [21]. The N:P ratios serve as crucial indicators of plant nutrient limitations [24], and the C:N ratio typically reflects a plant’s capacity to absorb various nutrients and assimilate carbon [25]. The composition, distribution, and relationship of C, N, and P in plants, along with their relationship to external environmental factors, collectively determine the nutritional status, growth, and development processes of plants [26]. Studying these factors can help elucidate the utilization and adaptation strategies of organisms to nutrients [27].

Currently, most research on Chinese fir has focused on asexual line selection and planting management. However, there are limited investigations into the roles of N, P, and K fertilizers in the nutrient cycle and the ecological stoichiometric characteristics of Chinese fir. Hence, this study primarily analyzed different seed-setting characteristics and pruned Chinese fir mother plants. Through the sampling and nutrient element measurement of pruned dwarf Chinese fir clones, this study explored the reasons for seed-setting differences after pruning. Additionally, fertilization experiments were conducted on pruned dwarf Chinese fir clones with different seed-setting characteristics to investigate the impact of fertilization treatments on the ecological stoichiometric characteristics of C, N, and P in Chinese fir needles, as well as the correlations among these characteristics and various seed-setting traits. To identify suitable formulas for enhancing production, this study addressed the issues of low numbers of female flowers, cone yield, and seed quality in pruned dwarf Chinese fir, providing a theoretical foundation for managing pruned dwarf seed orchards, practical technical support, and management insights for Chinese fir seed orchard development.

## 2. Materials and Methods

### 2.1. Overview of the Experimental Site

The experimental area is situated at the Yangkou State-owned Forest Farm in Fujian Province, characterized by a longitude of 117.902773° and latitude of 26.823542°, serving as the central production hub for Chinese fir. Established in 1956, the forest farm spans a total operating area of 4133 hm^2^ and harbors an existing forest stock of 590,000 m^3^. Nestled in the low hills of the Wuyi Mountain branch, the germplasm resource bank primarily comprises red soil featuring a fertile and deep soil layer. The experimental site, with a slope gradient ranging from 25° to 30°, faces southeast, with a soil layer thickness of 30–50 cm and a humus layer thickness of 10 cm. The total N content of the soil is 1.15 g/kg, the total P content is 0.39 g/kg, and the total K content is 7.71 g/kg. The herb layer beneath the forest is predominantly populated by *Dicranopteris linearis*, and *Adinandra millettii* dominates the shrub layer. The subtropical climate has an annual average temperature of 18.5 °C, an average annual precipitation of 1880 mm, and an extensive frost-free period lasting up to 305 d.

### 2.2. Experimental Materials and Methods

The experimental materials were sourced from the 4th-generation germplasm resource library of Chinese fir at Yangkou Forest Farm, located in Shunchang County, Nanping City, Fujian Province, and consisted of 608 clones. These clones were rooted in February 2015 and grafted in March–April 2016 and February–March 2017, respectively. The establishment of the germplasm resource library involved manual strip soil preparation, with a bandwidth of 1.2 m; the hole digging specification was 60 cm × 40 cm × 40 cm; and technical measures included 3 m × 3 m plant spacing and row spacing.

Based on the overall seed-setting characteristics of each clone in the germplasm resource library, nine cut-dwarfed Chinese fir clones with consistent growth potential and without pests or diseases were selected from Area 7 and Area 8. Clones No. 171, 178, and 281 were categorized as high-yield clones (Group A); clones No. 199, 244, and 307 were categorized as middle-yield clones (Group B); and clones No. 50, 246, and 324 were categorized as low-yield clones (Group C). Ten plants were selected from each clone as experimental subjects. There were a total of 90 experimental trees. In July 2021, one branch from each of the east, south, west, and north directions of the canopy periphery exhibiting similar growth conditions was selected for sampling. Subsequently, the needles were removed from these branches. The needles were placed in an icebox and transported to the laboratory. They were then dried at 105 °C for 30 min, followed by constant mass drying at 80 °C, and sieved after crushing. After thorough mixing, the samples were sealed in bags for testing. The C content in *Cunninghamia lanceolata* needles was determined using the K dichromate oxidation-external heating method. The N content was determined by the Kjeldahl method using a Kjeldahl nitrogen analyzer (KT—KS51, Xiamen Kelite Precision Instrument Co., Ltd., Xiamen, China). The P content was determined by the molybdic acid colorimetry method using a spectrophotometer (LAMBDA 750S, PERKINELMER, Massachusetts, MA, USA). The K content was determined by the flame atomic absorption spectrophotometry method using a flame spectrophotometer (420 Dual—channel, Cole Parme, Chicago, IL, USA) [28].

The N content was determined by the Kjeldahl method using a Kjeldahl nitrogen analyzer (KT—KS51, Xiamen Kelite Precision Instrument Co., Ltd.). The P content was determined by the molybdic acid colorimetry method using a spectrophotometer (LAMBDA 750S, PERKINELMER, Massachusetts, MA, USA). The K content was determined by the flame atomic absorption spectrophotometry method using a flame spectrophotometer (420 Dual—channel, Cole Parme, Chicago, IL, USA)

Since there was no variation in N content in the cut-off dwarf clone’s nutritional analysis, N was fixed at 10,000 mg/L in the fertilization design plan. An L9 (3^3^) orthogonal array was adopted. This array included three factors: phosphorus (P), potassium (K), and gibberellin (GA_3_), with each factor having three levels (P: 800, 1300, and 1800 mg/L; K: 1500, 2000, and 2500 mg/L; GA_3_: 50, 100, and 150 mg/L). The fertilizers used in the test were urea (containing 46% N), calcium superphosphate (containing 48% P_2_O_5_), and potassium chloride (containing 60% K_2_O). Additionally, each clone was subjected to a control treatment with distilled water (Table 1). The foliar fertilizer treatments were carried out on 2 June, 9 June, and 16 June in 2021, and again on 2 June, 9 June, and 16 June in 2022, corresponding to the cone growth and flower bud differentiation stages, respectively. Foliar spraying was carried out in the morning when the humidity was high and the wind was extremely gentle. The fertilizers and hormones required for each treatment were poured into a beaker, dissolved with a small amount of alcohol, and thoroughly stirred and diluted with a glass rod, then the volume was adjusted to 1000 mL. To enhance the nutrient absorption capacity of the needles and the adsorption capacity of the fertilizer solution, 0.15 mL of an agricultural organosilicon penetrant (ethoxylated polytrisiloxane) with a concentration of 0.1 g/L was added to the spraying solution. The fertilizer solution was filled into a pressure sprayer, and the front and back sides of the needles were sprayed evenly to ensure thorough spraying until the needles were covered with water droplets without dripping.

One month after the fertilization and one month after the cone harvesting of the same year, specifically in July and November 2021 as well as July and November 2022, branches were collected from the experimental trees, and the contents of C, N, P, and K in them were determined. The surveys of female and male coniferous flower quantities were conducted in March 2022 and March 2023. Additionally, in November 2021 and November 2022, the cones from the experimental trees were harvested. The total number of cones (pieces) was counted. Five cones were selected from each treatment, and the experiment was repeated six times. Statistical analyses were conducted on fresh cone weight (g), total seed weight (mg), seed hundred-seed weight (mg), seed type (plump seeds, astringent grains, and empty grains), cone size (mm), and seed size (mm), seed germination rate (GP,%), and seed germination index (GI) based on groups A, B, and C.

$$GP\left(\%\right)=\frac{N_{n}}{N}\times 100\%$$

GP represents the seed germination rate; N_n_ represents the number of germinated seeds; and N represents the total number of seeds.

$$GI=\sum_{i-1}^{n}\frac{Gi}{Di}$$

Gi represents the number of seeds that germinate on the i-th day; Di represents the corresponding number of days for germination; and n represents the total number of days of the germination test.

The experimental data are as follows: N content in November 2021 (N1), K content in November 2021 (K1), P content in November 2021 (P1), C:N in November 2021 (C/N1), N:P in November 2021 (N/P1), N content in July 2022 (N2), K content in July 2022 (K2), P content in July 2022 (P2), C:N in July 2022 (C/N2), N:P in July 2022 (N/P2), N content in November 2022 (N3), K content in November 2022 (K3), P content in November 2022 (P3), C:N in November 2022 (C/N3), N:P in November 2022 (N/P3), female cone flower in 2022 (F1), male cone flower in 2022 (M1), female cone flower in 2023 (F2), and male cone flower in 2023 (M2).

### 2.3. Statistical Analysis

The data underwent processing and analyses using the Excel software and R (version 4.1.1). The normality distribution verification, homogeneity testing, and one-way analysis of variance were conducted using R (version 4.1.1). A comprehensive scoring method was employed for comprehensive evaluation.

## 3. Results

### 3.1. Comparison of Nutritional Conditions of Chinese Fir Clones with Different Seed-Setting Characteristics After Cutting

In plant nutritional chemical diagnosis, research on total N content is the earliest and most comprehensive, effectively reflecting plant N status. N, known as the “element of life”, is crucial as the main constituent of protein and is pivotal in plant life processes. N applications significantly affect Chinese fir stem and leaf growth and development, closely correlating with yield [29]. Moreover, the carbon–nitrogen ratio in plants is intricately linked to their reproductive and nutritional growth. Higher ratios signify a shift toward reproductive growth, whereas lower ratios indicate nutritional growth.

**Figure 1:**
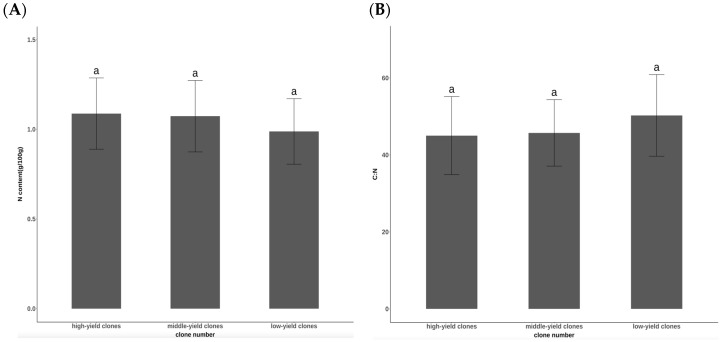
Comparison of (**A**) N content and (**B**) C:N ratios in different seed-setting characteristics of cut-off dwarf Chinese fir clones. Lowercase letters indicate that the clones with different seed-setting characteristics are significantly different at the 0.05 significance level (*p* < 0.05); same below.

As depicted in Figure 1A, there were no statistically significant variations in leaf N content among the different seed-setting characteristics of the *Cunninghamia lanceolata* clones (*p* = 0.281). The average N content of the high-yield clones of cut-off dwarfing Chinese fir clones was 1.09 g/100 g, whereas that of the middle-yield clones was 1.07 g/100 g. Conversely, the average N content of the low-yield clones was 0.98 g/100 g.

There was no significant difference in the C:N ratio among the three types of seed-setting characteristics of the clones (*p* = 0.247). The C:N ratios for the high-yield clones, middle-yield clones, and low-yield clones were 45.03, 45,73, and 50.28, respectively (Figure 1B).

**Figure 2:**
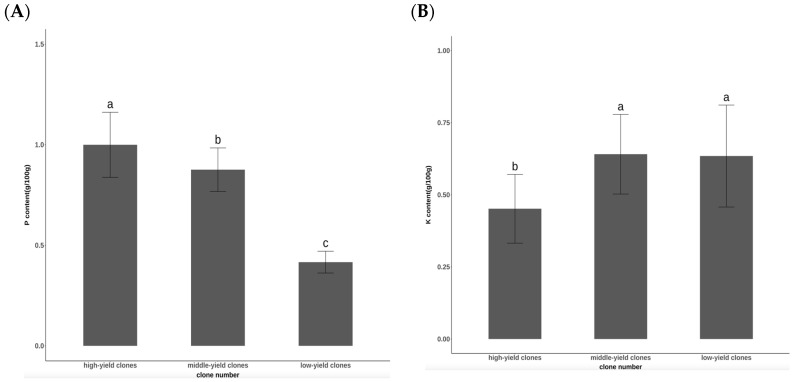
Comparison of (**A**) P and (**B**) K contents in different seed-setting characteristics of cut-off dwarf Chinese fir clones.

As shown in Figure 2A, significant differences in the P content among the different seed-setting characteristics of Chinese fir clones were observed after top-cutting and dwarfing (*p* = 0.0000208). The average P content for the high-yield clones was 0.99 g/100 g, for the middle-yield clones was 0.88 g/100 g, and for the low-yield clones was 0.41 g/100 g.

Following pruning and dwarfing, substantial differences in K content were evident among the various seed-setting characteristics of the *Cunninghamia lanceolata* clones (*p* = 0.000155) (Figure 2B). The average K content was 0.45 g/100 g for clones with a higher seed setting, 0.64 g/100 g for clones with a medium seed setting, and 0.63 g/100 g for clones with a lower seed setting.

### 3.2. Impact of Foliar Fertilization on the N, P, and K Contents of Chinese Fir Needles

(1) Impact of foliar fertilization on total N in the needles of Chinese fir

**Figure 3:**
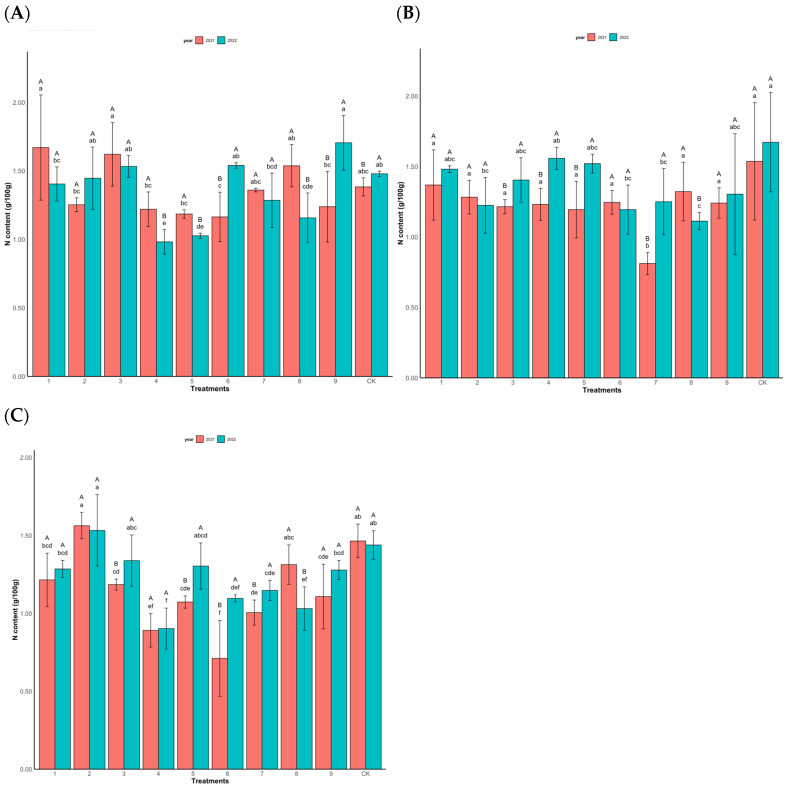
Changes in the total N content (g/100 g) of Chinese fir needles under different fertilization levels. (**A**) is high-yield clones (Group A), (**B**) is middle-yield clones (Group B), and (**C**) is low-yield clones (Group C). Lowercase letters indicate significant differences at the 0.05 significance level among different treatments in the same year (*p* < 0.05); capital letters indicate significant differences at the 0.05 significance level for the same treatment across different years (*p* < 0.05). Same below.

In Figure 3, group A displays notable variations in N content among different treatments for the high-yield clones in both 2021 and 2022 (*p* = 0.0002). In 2021, the total N content in Chinese fir needles under treatments T1 and T3 surpassed that of the control with water treatment, with the highest N content observed in treatment T1 (N 10,000 mg/L:P 1300 mg/L:K 2000 mg/L:GA_3_ 100 mg/L). Conversely, treatment T6 exhibited significantly lower N content compared to the control, featuring the formulation N 10,000 mg/L:P 1800 mg/L:K 1500 mg/L:GA_3_ 100 mg/L. In 2022, treatment T4 recorded the lowest total N content at 0.98 g/100 g, employing the formulation N 10,000 mg/L:P 800 mg/L:K 2000 mg/L:GA_3_ 150 mg/L, whereas treatment T9 exhibited the highest total N content at 1.71 g/100 g, utilizing the formula N 10,000 mg/L:P 800 mg/L:K 1500 mg/L:GA_3_ 50 mg/L.

In Figure 3B, the N content significantly affects the growth of the middle-yield clones (*p* = 0.006). In 2021 and 2022, the total N content of all the treatments was lower than the control. The total N concentration of T7 processed in 2021 was the lowest, at 0.81 g/100 g. In 2022, T8 had the lowest total N content at 1.11 g/100 g.

Figure 3C depicts the fluctuations in the N content of the low-yield clones under various treatments in both 2021 and 2022. In 2021, treatment T2 exhibited the highest total N content at 1.56 g/100 g, formulated as N 10,000 mg/L:P 1300 mg/L:K 2500 mg/L:GA_3_ 50 mg/L; conversely, treatment T6 had the lowest total N content at 0.71 g/100 g. In November 2022, treatment T2 maintained the highest total N content of 1.53 g/100 g, whereas treatment T4 displayed the lowest total N content of 0.90 g/100 g. Notably, there was minimal variation in the total N content between the two years.

(2) Impact of foliar fertilization on total P in the needles of Chinese fir

**Figure 4:**
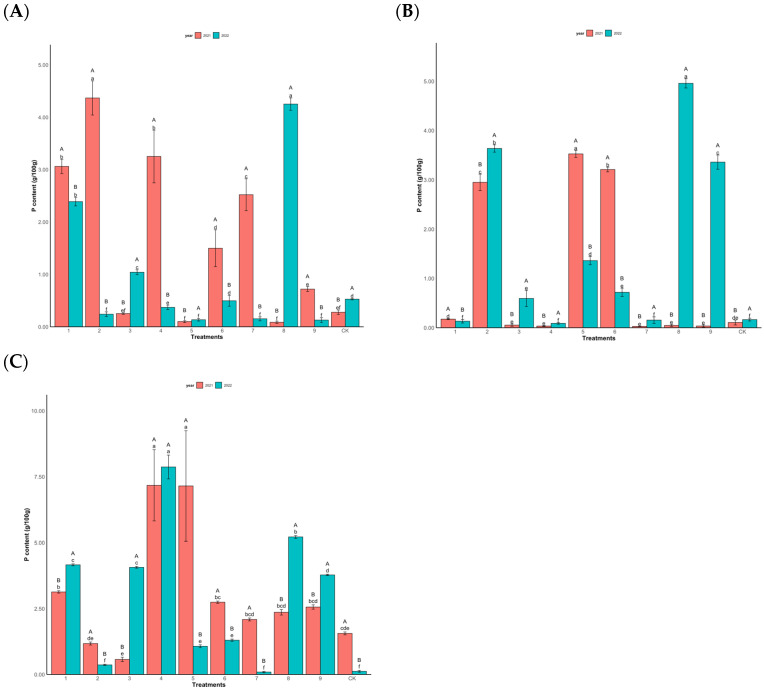
Changes in total P content (g/100 g) of Chinese fir needles under different fertilization levels. (**A**) is high-yield clones (Group A), (**B**) is middle-yield clones (Group B), and (**C**) is low-yield clones (Group C).

In Figure 4, significant differences were observed in the needle contents of the different clones with distinct fruiting characteristics following various fertilization treatments (*p* < 0.001). In 2021, among the high-yield clones, the T2 treatment demonstrated the highest total phosphorus content at 4.37 g/100 g, with only the T8 treatment showing a lower value (0.08 g/100 g) than the control. In 2022, the T8 treatment recorded the highest phosphorus content (4.27 g/100 g) among the high-yield clones (Figure 4A). For the middle-yield clones in 2021, the T5 treatment exhibited the highest phosphorus content (3.53 g/100 g), whereas the T4 and T7 treatments displayed the lowest values (0.03 g/100 g). In 2022, the T2, T3, T5, T6, T8, and T9 treatments all exceeded the control group, with the T8 treatment achieving the highest phosphorus content (4.95 g/100 g) and T4 treatment showing the lowest (0.09 g/100 g) among the middle-yield clones (Figure 4B). Regarding the low-yield clones, the T4 treatment displayed the highest phosphorus content (7.17 g/100 g) in 2021, while the T3 treatment had the lowest (0.56 g/100 g). In 2022, the T4 treatment maintained the highest phosphorus content (7.87 g/100 g), whereas the T7 treatment recorded the lowest (0.09 g/100 g) among the low-yield clones (Figure 4C).

(3) Impact of foliar fertilization on total K in the needles of Chinese fir

**Figure 5:**
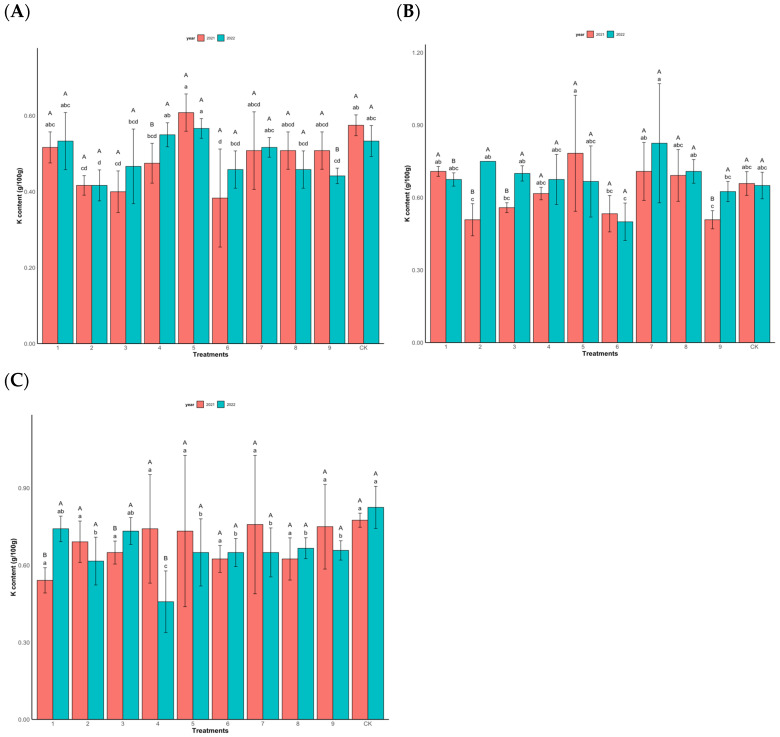
Changes in total K content (g/100 g) of Chinese fir needles under different fertilization levels. (**A**) is high-yield clones (Group A), (**B**) is middle-yield clones (Group B), and (**C**) is low-yield clones (Group C).

In Figure 5, there is a significant difference in the potassium content in the needles after different treatments (*p* < 0.001). In 2021, treatment T5 demonstrated the highest K concentration (0.61 g/100 g) among the high-yield clones. This trend persisted in 2022, where T5 maintained the highest K content (0.55 g/100 g) under the formulation N 10,000 mg/L, P 1800 mg/L, K 2000 mg/L, and GA_3_ 50 mg/L (Figure 5A). The middle-yield clones showed dynamic responses to treatments. In 2021, T5 recorded the highest K content (0.78 g/100 g), while T2 and T9 exhibited significantly lower values (0.51 g/100 g). By 2022, T7 emerged as the top-performing treatment (0.83 g/100 g) (Figure 5B). The low-yield clones displayed contrasting temporal patterns. In 2021, T7 achieved the highest K content (0.76 g/100 g), whereas T1 showed the poorest accumulation (0.54 g/100 g). This pattern reversed in 2022, with T1 demonstrating the highest K accumulation (0.74 g/100 g) and T4 registering the lowest (0.46 g/100 g) (Figure 5C).

### 3.3. Effects of Foliar Fertilization on the Ecological Stoichiometric Characteristics of C, N, and P in Chinese Fir Needles

**Figure 6:**
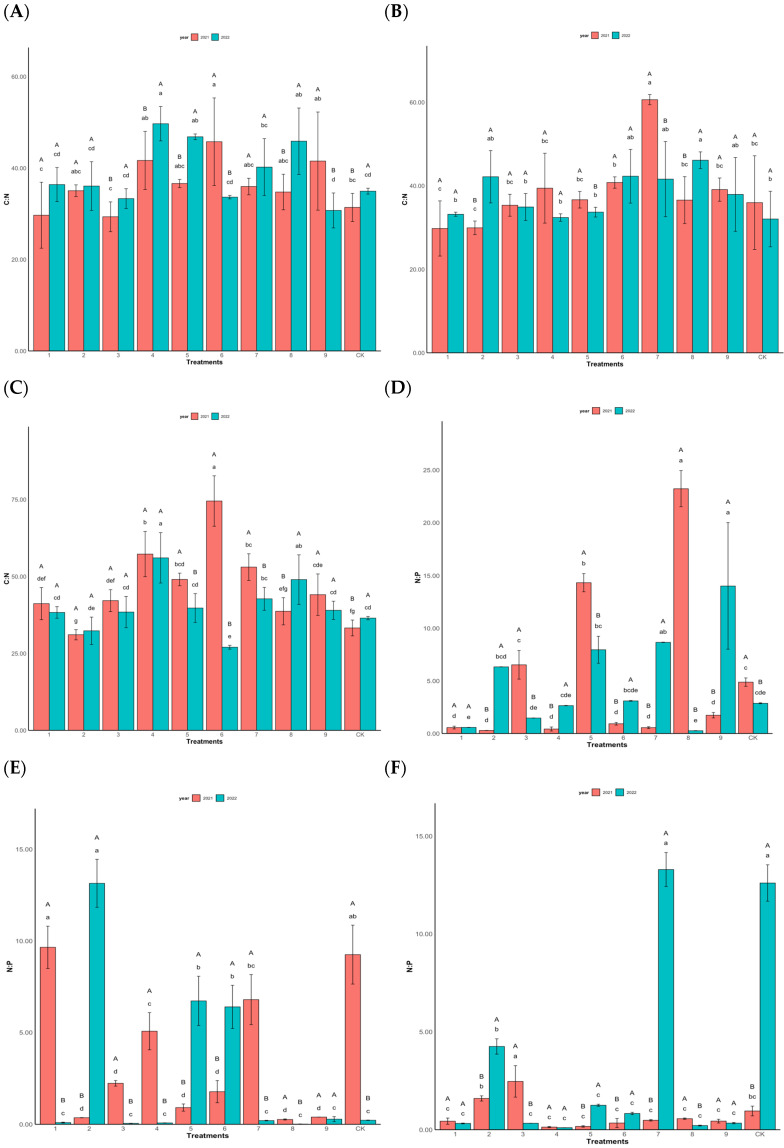
C, N, and P stoichiometric characteristics of Chinese fir needles under different fertilization levels. (**A**) is the C:N ratio of high-yield clones; (**B**) is the C:N ratio of middle-yield clones; (**C**) is the C:N ratio of low-yield clones; (**D**) is the N:P ratio of high-yield clones; (**E**) is the N:P ratio of middle-yield clones; (**F**) is the N:P ratio of low-yield clones.

The C:N ratio significantly influenced the reproductive and vegetative growth of the plants. In 2021, the high-yield clones exhibited C:N ratios ranging from 29.35 to 45.77, with the treatment T6 recording the highest C:N ratio. Conversely, T3 had the lowest C:N ratio. In 2022, treatment T4 had the highest C:N ratio of 49.70, whereas treatment T9 had the lowest C:N ratio of 30.73 (Figure 6A). Among the middle-yield clones in 2021, the C:N ratios ranged from 29.78 to 60.64, with T7 recording the highest ratio and T1 the lowest. In 2022, treatment T8 exhibited the highest C:N ratio of 46.13, whereas the control had the lowest ratio of 32.05 (Figure 6B). Regarding the low-yield clones in 2021, T6 displayed the highest C:N ratio of 73.42, whereas T2 had the lowest ratio of 31.09. In 2022, T4 had the highest C:N ratio of 56.09, while treatment T6 had the lowest ratio of 27.04. The average C:N ratio in 2021 was 47.16, which decreased to 36.56 in 2022, indicating a declining trend possibly attributed to the weakened reproductive growth of the low-yield clones and a decrease in male or female cones (Figure 6C).

The critical N:P ratio indicates the internal structure and functional status of plants, facilitating the assessment of the nutrient supply required for plant growth [30]. Among the high-yield clones in 2021, treatment T2 displayed the lowest N:P ratio of 0.28, whereas treatment T8 had the highest N:P ratio of 23.22. Treatment T5 had an N:P ratio of 14.31. In 2022, treatment T8 showed the lowest N:P ratio of 0.27, whereas treatment T9 had the highest N:P ratio of 14.01 (Figure 6D). Among the middle-yield clones, treatment T8 had the lowest N:P ratio (0.26) in 2021, whereas treatment T1 had the highest N:P ratio of 9.60. In 2022, T8 exhibited the lowest N:P ratio of 0.01, whereas T2 had the highest N:P ratio of 13.11. The N:P ratios of treatments and controls in both 2021 and 2022 were below 14.00 (Figure 6E). Among the low-yield clones, treatment T4 had the lowest N:P ratio (0.13) in 2021, whereas treatment T3 had the highest N:P ratio of 2.46. In 2022, T4 showed the lowest N:P ratio of 0.11, whereas T7 had the highest N:P ratio of 13.29. The N:P ratios of treatments and controls in both 2021 and 2022 were below 14.00 (Figure 6F).

### 3.4. Analysis of Foliar Fertilization on the Yield and Quality of Chinese Fir Cones

(1) Effect of foliar fertilization on cone number of clones with different seed-setting types

**Figure 7:**
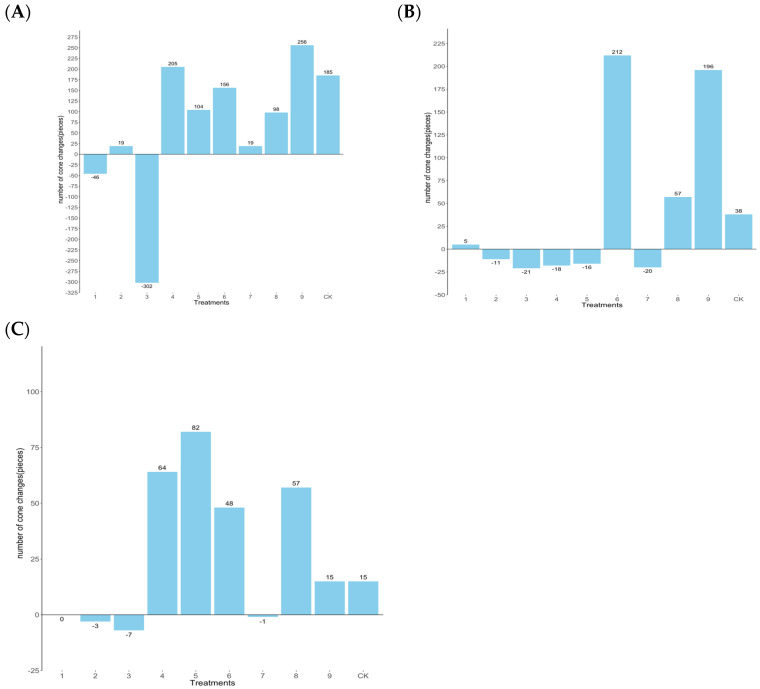
Growth of the number of Chinese fir cones in 2022 under different levels of fertilization. (**A**) is high-yield clones (Group A), (**B**) is middle-yield clones (Group B), and (**C**) is low-yield clones (Group C).

As the initial fertilization occurred in July 2021, coinciding with the onset of cone development, the analysis primarily focused on cone quantity in 2022. Among the high-yield clones (Figure 7A), treatment T3 decreased by 302 cones compared to 2021, while treatment T1 experienced a reduction of 46 cones. Conversely, treatment T9 showed an increase of 256 cones, and treatment T4 showed an increase of 205 cones compared to 2021. For the middle-yield clones (Figure 7B), treatments T2, T3, T4, T5, and T7 recorded decreases of 11, 21, 18, 16, and 20 cones, respectively, compared with 2021. On the other hand, treatments T1, T6, T8, and T9 exhibited increases of 5, 212, 57, and 196 cones, respectively, indicating superior performance by treatment T6. Among the low-yield clones (Figure 7C), treatments T1, T2, T3, and T7 showed marginal decreases of 0, 3, 7, and 1 cones, respectively. Conversely, treatments T4, T5, T6, T8, and T9 showed increases in 64, 82, 48, 57, and 15 cones, respectively, with treatment T5 demonstrating better performance. Excessive P fertilizer in treatment T1 compared to treatments T4 and T9 may contribute to the reduction in cone quantity in Chinese fir clones after stem cutting and dwarfing.

(2) Correlation analysis between the number of Chinese fir cones with different fruiting characteristics and N, P, and K.

**Figure 8:**
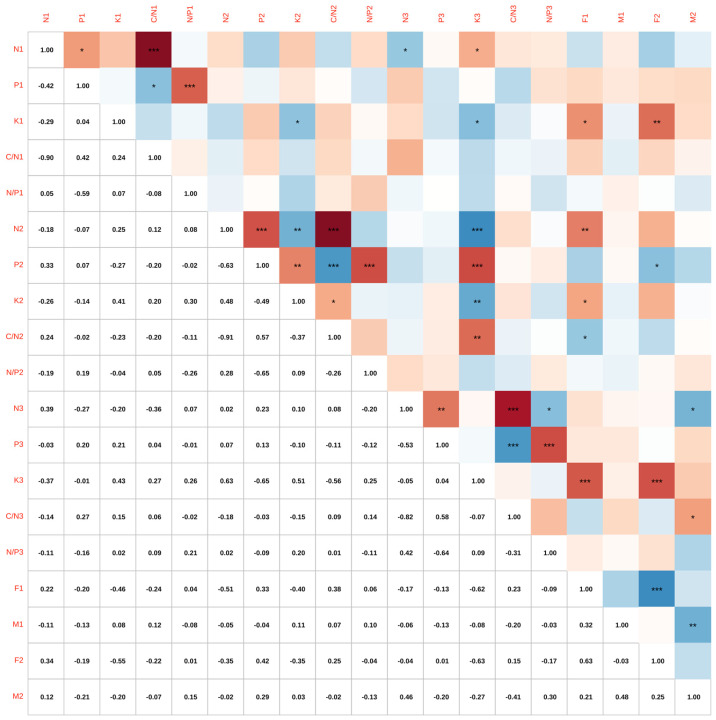
Correlation analysis heatmap of the number of female and male cones and N, P, and K contents in Chinese fir with different cone numbers. Different colors represent the magnitudes of the correlation coefficients among various traits. The number represents the correlation coefficient between traits. The asterisk (*) represents the significance level. Significance levels: *, *p* < 0.05; **, *p* < 0.01; ***, *p* < 0.001.

The correlation analysis of the number of female and male cones, and the N, P, and K contents in the needles of three types of Chinese fir clones with high, medium, and low seed production are shown in Figure 8. There are varying degrees of correlation between the traits. From Figure 8, it can be concluded that in April 2022, the number of female cones had a significant negative correlation with the K content in November 2021 (*p* < 0.05, r = −0.46), a very significant negative correlation with the N content in July 2022 (*p* < 0.01, r = −0.51), a significant negative correlation with the K content in July 2022 (*p* < 0.05, r = −0.40), a significant positive correlation with the C:N ratio in July 2022 (*p* < 0.05, r = 0.38), and an extremely significant negative correlation with the K content in November 2022 (*p* < 0.001, r = −0.62). In April 2023, the number of female cones showed a significant positive correlation with the P content in July 2022 (*p* < 0.05, r = 0.42), a very significant negative correlation with the K content in November 2021 (*p* < 0.01, r = −0.55), and an extremely significant negative correlation with the K content in November 2022 (*p* < 0.001, r = −0.63). In April 2023, the number of male cones had a significant positive correlation with the N content in November 2022 (*p* < 0.05, r = 0.46) and a significant negative correlation with the C:N ratio in November 2022 (*p* < 0.05, r = −0.41). The number of female cones in April 2022 showed an extremely significant positive correlation with the number of female cones in April 2023 (*p* < 0.001, r = 0.63). The number of male cones in April 2022 showed a very significant positive correlation with the number of male cones in April 2023 (*p* < 0.01, r = 0.48).

(3) Path analysis of N, P, and K content in China fir needles on cone quantity

The number of cones is an important indicator of the fruiting ability of Cunninghamia. Using the number of cones in 2022 (Y) as the dependent variable, and the N content in November 2021 (X1), P content in November 2021 (X2), K content in November 2021 (X3), C/N ratio in November 2021 (X4), N/P ratio in November 2021 (X5), N content in July 2022 (X6), P content in July 2022 (X7), K content in July 2022 (X8), C/N ratio in July 2022 (X9), and N/P ratio in July 2022 (X10) as independent variables, a stepwise regression analysis was conducted. Using the backward elimination method, the regression equation with 10 indicators was refined by successively selecting the independent variables. The results showed that the P content in November 2021 (X2), N content in July 2022 (X6), P content in July 2022 (X7), C/N ratio in July 2022 (X9), and N/P ratio in July 2022 (X10) were the main factors influencing the number of cones of China fir, with significant correlations.

**Figure 9:**
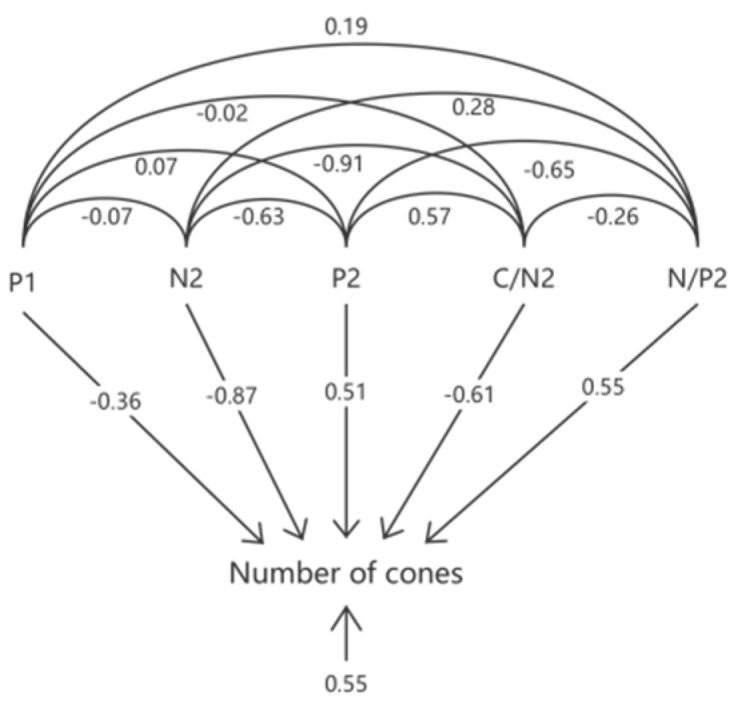
Path analysis of N, P, and K content in China fir needles on cone quantity.

Path analysis was performed using the five indicators selected by the stepwise regression, as shown in Figure 9. The P content in November 2021 (X2), N content in July 2022 (X6), P content in July 2022 (X7), C/N ratio in July 2022 (X9), and N/P ratio in July 2022 (X10) contributed differently to the number of cones in 2022 (Y), showing either positive or negative effects. The factor with the greatest positive direct influence on the number of cones was the N/P ratio in July 2022 (X10), with a direct effect of 0.55, while the factor with the greatest negative direct influence was the N content in July 2022 (X6), with a direct effect of −0.87. The direct effects of the nutritional indicators on the germination rate, in descending order, were as follows: N/P ratio in July 2022 (X10) > P content in July 2022 (X7) > P content in November 2021 (X2) > C/N ratio in July 2022 (X9) > N content in July 2022 (X6).

(4) Comprehensive evaluation of foliar fertilization on needle nutrition and fruiting

The primary objective of fertilizing the clones with various seed-setting characteristics was to enhance the seed yield and quality of Chinese fir stump dwarf seed orchards. Based on the preceding analysis, different fertilization treatments yielded varying effects on pertinent indicators, such as needle nutrition, cone quantity, seed quality, and flower quantity across clones with distinct seed-setting types. To provide an objective assessment of the nine treatments, an analysis was conducted on needle nutrition, cone count, seed quality, and flower quantity of cut-off dwarf clone mother trees exhibiting diverse seed-setting characteristics. A comprehensive scoring method was employed to assess needle N, C:N, K, and P contents; cone count; flower quantity; seed germination rate; 100-seed weight; cone phenotype; and seed phenotype across different treatments for the current year. The comprehensive score reflected seed yield and quality following treatment. After the July 2021 treatment, the high-yield clone treatment T5 achieved the highest score, while treatment T7 scored the lowest. Similarly, for the middle-yield clones, treatment T3 attained the highest score, with treatment T6 scoring the lowest. The low-yield clone treatment T3 received the highest score, whereas treatment T7 scored the lowest. Following the July 2022 treatment, the high-yield clone treatment T8 obtained the highest score, in contrast to CK, which scored the lowest. Among the middle-yield clones, treatment T8 received the highest score, while treatment T6 received the lowest score. Finally, for the low-yield clones, treatment T4 had the highest score, with treatment CK obtaining the lowest (Table 2). Owing to fluctuations in cone numbers between 2021 and 2022, the scores varied for the two years. Therefore, in practical fertilizer applications, adjustments to the fertilizer formula should align with the growth status of Chinese fir plants to optimize yield.

## 4. Discussion

### 4.1. Comparison of Nutrition Status of Chinese Fir Clones with Different Seed-Setting Characteristics After Cutting

In plant nutritional chemical diagnosis, research on the total N content is the earliest and most comprehensive, effectively reflecting plant N status. N, known as the “element of life”, is crucial as the main constituent of protein and is pivotal in plant life processes. N applications significantly affect Chinese fir stem and leaf growth and development, closely correlating with yield [29]. Moreover, the carbon–nitrogen ratio in plants is intricately linked to their reproductive and nutritional growth. Higher ratios signify a shift toward reproductive growth, whereas lower ratios indicate nutritional growth. The study on the third-generation seed orchard of *Cunninghamia lanceolata* found that the N and P content in the needles of high-yielding mother trees was higher than that of low-yielding mother trees, while the K content was higher in low-yielding mother trees compared to high-yielding ones. However, the differences in the content of these elements were not significant [31]. This experiment found that the N content of highly productive mother trees showed no significant difference compared to the vegetative clones with medium or low productivity, but the P content was significantly higher than that of the medium- and low-productivity clones, while the K content was significantly lower, which is inconsistent with the findings of Liu Wenfei’s research [31].

The nutritional and reproductive growth of the Chinese fir involved a multifaceted interplay of internal and external factors, making it a complex process. Mineral nutrition plays a vital role in forest tree growth. Following stem cutting and dwarfing, the Chinese fir clones exhibited notable variability, with some yielding over 200 cones, whereas others produced only one. This discrepancy significantly affected the seed yield of Chinese fir seed orchards. Hence, investigating nutrient regulation after stem cutting and dwarfing was imperative to provide a theoretical framework for nutrient fertilization. This study revealed no significant variance in the total N and C:N ratios across different clonal, consistent with the findings from *Pinus koraiensis* studies [32].

In this study, it was found that the P fertilizer significantly affected Chinese fir by stimulating flower bud differentiation, accelerating flowering and fruiting, and enhancing cone quality. Consequently, there was a higher demand for P during the early stages of Chinese fir growth. This is consistent with the research results of *Pinus massoniana* [10,33]. A fertilization investigation was carried out on *Zanthoxylum bungeanum*, the results indicated that the application of N, P, and K fertilizers significantly augmented the flower bud differentiation rate of *Zanthoxylum bungeanum* [34]. In the present study, it was discovered that the early P accumulation profoundly affected later flower bud differentiation and cone quality in Chinese fir. The clones with greater seed-setting tendencies accumulated more P, which was utilized for cone development and seed maturation. In subsequent experiments, the application of P fertilizer was employed to promote the differentiation of female flower buds in Chinese fir. Increased P content could facilitate nutrient accumulation in the flower buds. These findings are in accordance with the research outcomes of *Zanthoxylum bungeanum*, suggesting a potential commonality in the role of P in the reproductive processes of these two species.

K significantly enhanced the quality and yield of *Cunninghamia lanceolata* seeds. Given that sampling occurred in July, when cone development commenced, K was crucial for cone growth. Therefore, the K from the branches supported the cone and seed development, resulting in reduced K content in the needles. K plays a crucial role in carbohydrate synthesis, particularly during the later stages of flower bud differentiation; this is consistent with the research results of *Camellia oleifera* [35]. This investigation revealed that the high-yield clones exhibited lower K levels than the low-yield clones. Some scholars have conducted research on *Pistacia chinensis* and also obtained consistent results [36]. This difference may stem from the heightened demand for K during cone growth and seed development in July. Therefore, to enhance the quality of Chinese fir cones and seeds, it is advisable to supplement them with specific K fertilizers during their development.

### 4.2. Effects of Fertilization Treatments on the Ecological Stoichiometry Characteristics of Chinese Fir Needles

In this study, the total N content exhibited significant variations across different years and seed-setting traits, consistent with the findings in *Pinus koraiensis* research [37]. Specifically, the total N content of the high-yield clones in 2021 was 1.37 g/100 g, and in 2022, it was 1.34 g/100 g. For the middle-yield clones, the content was 1.20 g/100 g in 2021 and 1.39 g/100 g in 2022, while for the low-yield clones, it was 1.12 g/100 g in 2021 and 1.22 g/100 g in 2022. Given the fixed concentration of N fertilizer used in this experiment, the interaction among various elements is essential for optimizing N fertilizer efficiency [38]. During the peak growth period of Chinese fir, spanning from May to November of each year, substantial energy was required for flower bud differentiation, spike emergence, cone expansion, and seed matter accumulation. As cone development was initiated, N absorption gradually intensified, catering to the needs of cones and seeds. Consequently, the clones with higher branch counts needed more N for reproductive growth.

P demonstrates significant mobility within plants, and its accumulation and distribution across various organs play pivotal roles in plant growth and in enhancing fertilizer utilization efficiency [39]. The application of P fertilizer notably increases P levels in Chinese fir, stimulating female flower differentiation, and thereby enhancing yield; this is consistent with the research results of *Malus pumila* [9] and *Castanea mollissima* [40]. In a two-year foliar spraying comparison, the high-yield clones treated with T2, T8, and T9 exhibited a 35.12% increase in the total P content compared with the control. Among these treatments, T8 and T9 had P concentrations of 800 mg/L, whereas T2 had 1300 mg/L. For the middle-yield clones, treatments T6, T8, and T9 led to a 31.18% higher total P content compared to the control. The P concentration in treatments T8 and T9 was 800 mg/L, whereas that in treatment T6 was 1800 mg/L. In the low-yield clones, treatment T4 resulted in a 25.31% increase in the total P content compared to that of the control, with a concentration of 800 mg/L, indicating significant variations in P absorption capacity among the different clones. Chinese fir clones with robust cone-bearing capacity exhibit higher P levels in their needles, aligning with the substantial P requirement for female cone formation observed in *Pinus massoniana* development [33]. Nonetheless, excessive P fertilizer concentration can reduce the total P content in the needles of clones with robust cone-bearing abilities. Higher P levels can elevate the total P content in the needles of the middle-yield clones, whereas low-yield clones, which produce fewer female fruits, necessitate lower P fertilizer levels. Application rates of 800 mg/L or 1300 mg/L are economically viable and suitable for various stump-dwarfing Chinese fir clones with distinct fruiting characteristics. P plays a crucial role in regulating the ratio of male to female inflorescences in Chinese fir, notably boosting the number of female flowers [41]. Timely P supplementation, particularly during the reproductive growth phase of Chinese fir, is an effective strategy for enhancing female fertility and ensuring fruit production.

The application of K fertilizer enhanced the seed-setting ability of Chinese fir and increased the yield of cones. During critical growth stages, such as flowering and early fruit development, nutrient absorption by the root system may not adequately satisfy the morphological requirements of the aboveground organs [42]. In agricultural practice, foliar fertilization is a viable method for mitigating nutrient deficiencies in plants [43]. Following fertilization treatment, the total K content varied across clones between 2021 and 2022: the high-yield clone exhibited a total K content of 0.49 g/100 g in 2021 and 0.60 g/100 g in 2022, the middle-yield clone displayed 0.61 g/100 g in 2021 and 0.68 g/100 g in 2022, while the low-yield clone showed 0.65 g/100 g in 2021 and 0.60 g/100 g in 2022. Among the treatments, T5 applied to the high-yield clone demonstrated the highest total K content in needles, registering a K concentration of 2000 mg/L, whereas T7 applied to the middle-yield clone exhibited the highest total K content, with a K concentration of 2500 mg/L. The low-yield clone treatments T1 and T7 exhibited the highest total K content, with K concentrations of 2000 mg/L and 2500 mg/L, respectively. Higher concentrations of K fertilizer promoted the total K content in the needles of the cut-off dwarf Chinese fir clone, facilitating the differentiation of female flowers and cone growth in Chinese fir. This finding aligned with research outcomes observed in *Pinus koraiensis* [44].

Ecological stoichiometric ratio characteristics serve as indicators of plant growth, development, and nutrient supply. A higher leaf C:N ratio indicates an enhanced plant capacity to assimilate C while absorbing nutrient chemical elements, reflecting the efficiency of nutrient absorption and utilization. Numerous studies have demonstrated a strong correlation between elemental stoichiometric characteristics and plant growth rate [45]. In 2021, the fertilization of the high-yield clones resulted in higher C:N ratios than the control in all the treatments except T1 and T3, whereas for the middle-yield clones, only T1, T2, and T3 had lower C:N ratios than the control. Similarly, for the low-yield clones, only T2 showed a lower C:N ratio than that of the control. In 2022, the fertilization of the high-yield clones led to higher C:N ratios compared to the control in all treatments, except T3, T6, and T9. Conversely, for the middle-yield clones, the C:N ratios in all treatments exceeded those of the control. For the low-yield clones, except for treatments T2 and T6, the C:N ratio was lower than that of the control, whereas the remaining treatments exhibited higher ratios. The results of two consecutive years of experimentation indicated that the application of N fertilizer to Chinese fir leads to elevated C:N ratios in its needles, thereby enhancing the efficiency of nutrient absorption and utilization in the species [46].

The growth rate hypothesis suggests that plants with faster growth rates and organs with higher metabolic rates tend to exhibit lower N:P ratios with an increase in P content [47]. The N:P threshold hypothesis [48] suggests that leaf N:P ratios can indicate the limiting elements for plant growth. Typically, ratios below 14 indicate N limitation, whereas ratios above 16 indicate P limitation. Ratios between 14 and 16 generally indicate the limitations of both N and P. In 2021, treatment T5, applied to the high-yielding Chinese fir clones, exhibited N:P ratios between 14 and 16, whereas treatment T8 had ratios exceeding 16, and the remaining treatments had ratios below 14. In 2022, only treatment T9 had N:P ratios between 14 and 16, while the remaining treatments had ratios below 14, indicating that after most treatments, N largely constrained the growth of high-yielding Chinese fir clones. For the middle- and low-yield clones, after fertilization treatments in both 2021 and 2022, the N:P ratios for all the treatments were below 14, indicating that N primarily limited the growth of the middle- and low-yield clones of Chinese fir. Some current studies suggest that plant growth in subtropical evergreen forest areas is primarily limited by P [49,50,51], but our research findings are not in line with this view. This variation could result from diverse nutrient requirements among plant species for N and P nutrients or the relatively low N content present in the examined deep red soil [52]. Given the fixed N fertilizer amount used in this study, subsequent experiments should consider setting varied N concentrations to examine their distinct effects on Chinese fir growth.

### 4.3. Analysis of Seed Yield and Quality of Chinese Fir Treated with Fertilizer

In seed orchards, cone production significantly influences the seed yield. Increasing cone numbers and minimizing on- and off-year fluctuations are crucial for achieving consistently high yields. Tailored fertilization formulas should be applied to clonal plants with varying seed-setting characteristics. Treatment with T9 notably enhanced cone fruit production in high-yield clones. Similarly, treatment T6 increased cone fruit production in middle-yield clones by 4.5 times compared with the control. Treatment T5 proved the most effective in boosting cone fruit production in low-yield clones, achieving a 4.5-fold increase over the control.

Addressing the presence of tannin-like and empty seeds remains a significant concern for Chinese fir producers, who emphasize the importance of minimizing their occurrence. Seed features, such as size, reflect the internal nutrient content and influence seed dissemination and germination [53]. Employing suitable fertilization ratios can effectively enhance seed shape in cut-dwarfed asexual Chinese fir lines, thereby improving overall seed quality.

In conclusion, upon a comprehensive assessment of relevant indicators among cut-off dwarfing Chinese fir clones with varying seed-setting characteristics following different treatments, disparities in clone scores were observed. Therefore, tailored fertilization approaches are essential for meeting specific requirements. Guiding fertilizer application based on the characteristics of cut-off dwarfing Chinese fir clones, diverse growth stages (including cone development, seed endosperm filling, and clone seed-setting characteristics), and phenology and their interrelations is imperative to achieve optimal fertilizer utilization and ensure high and consistent yields in seed orchards.

## 5. Conclusions

This study examined Chinese fir clones with diverse seed-setting characteristics and investigated nutritional indicators to explore their relationships. Following stem cutting, the Chinese fir clones were categorized into high-, medium-, and low-yield groups based on seed setting. They underwent the foliar spraying of fertilizers and hormones, with a focus on comparing the C, N, P, and K contents and ecological stoichiometry among the clones after fertilization. This study aimed to determine the appropriate fertilization ratio for providing a theoretical basis for enhancing the high and stable yield of Chinese fir after stem cutting and dwarfing. The study concluded the following:

(1) After pruning, the Chinese fir cones exhibited notable variations. In July, there were no significant differences in total N content and C:N ratio among the different seed-setting characteristics. However, the P content in the high-yield clones was markedly higher than that in the middle-yield and low-yield clones, while the K content was notably lower. This demonstrated the significance of July in cone and seed development, which required P and K supplementation. Attention to these indicators can be crucial to scientifically enhancing seed orchard yields.
(2) Fertilization could be a vital strategy for enhancing the nutritional growth of Chinese fir and increasing seed yield. Strategic fertilization not only conserved costs, but also boosted yields. To elevate Chinese fir seed yield post-stem cutting and dwarfing and to mitigate on-and-off-year fluctuations, various fertilizer and hormone ratios were designed through orthogonal design. The objective was to identify the optimal fertilization scheme for different seed-setting characteristics. For the high-yield clones, four formulations such as Treatment 5, Treatment 6, Treatment 8, and Treatment 9 can be applied, which can enhance the nutrient level of branches and increase the number of cones. For the middle-yield clones, three formulations like Treatment 3, Treatment 6, and Treatment 9 can be used to improve needle nutrition and the number of cones. For the low-yield clones, applying three formulations including Treatment 3, Treatment 4, and Treatment 5 can increase the nitrogen, phosphorus, and potassium contents by 18.54%, 36.57%, and 26.56%, respectively, and increase the number of cones by 82. Therefore, during fertilization, tailored formulas should be employed according to the specific seed-setting characteristics of Chinese fir and management objectives to effectively enhance yield.

## Figures and Tables

**Table 1:** Orthogonal experimental design table.

Treatments	N (mg/L)	P (mg/L)	K (mg/L)	GA_3_ (mg/L)
T1 (N_0_P_2_K_2_GA_3 2_)	10,000	1300	2000	100
T2 (N_0_P_2_K_3_GA_3 1_)	10,000	1300	2500	50
T3 (N_0_P_2_K_1_GA_3 3_)	10,000	1300	1500	150
T4 (N_0_P_1_K_2_GA_3 3_)	10,000	800	2000	150
T5 (N_0_P_3_K_2_GA_3 1_)	10,000	1800	2000	50
T6 (N_0_P_3_K_1_GA_3 2_)	10,000	1800	1500	100
T7 (N_0_P_3_K_3_GA_3 3_)	10,000	1800	2500	150
T8 (N_0_P_1_K_3_GA_3 2_)	10,000	800	2500	100
T9 (N_0_P_1_K_1_GA_3 1_)	10,000	800	1500	50
CK	0	0	0	0

**Table 2:** Comprehensive score of different fertilization treatments for clones with different types of seed-setting characteristics.

Treatments	Comprehensive Score					
	Fertilization Treatment in 2021			Fertilization Treatment in 2022		
	High-Yield Clone	Middle-Yield Clone	Low-Yield Clone	High-Yield Clone	Middle-Yield Clone	Low-Yield Clone
T1	0.2483	0.2306	0.2875	0.2597	0.2272	0.2609
T2	0.2812	0.2446	0.2866	0.2544	0.2804	0.2598
T3	0.2777	0.2967	0.2975	0.2409	0.2305	0.2599
T4	0.2841	0.2488	0.2192	0.4315	0.2608	0.2885
T5	0.2901	0.2608	0.2667	0.2098	0.2670	0.2779
T6	0.2866	0.2012	0.2879	0.2469	0.2617	0.2600
T7	0.2421	0.2492	0.2076	0.1552	0.2804	0.1746
T8	0.2838	0.2198	0.2914	0.4627	0.296	0.2598
T9	0.2842	0.2539	0.2826	0.1610	0.2849	0.2629
CK	0.2757	0.2760	0.2339	0.0753	0.2721	0.0772

## Data Availability

Data are contained within the article.
